# Contagion comeback: unravelling the measles outbreak across the USA

**DOI:** 10.3389/fpubh.2024.1491927

**Published:** 2024-12-18

**Authors:** Iman Muhammad Tahir, Vijay Kumar, Hanya Faisal, Anosh Gill, Vineeta Kumari, Haya Muhammad Tahir, Md Ariful Haque

**Affiliations:** ^1^Liaquat National Hospital and Medical College, Karachi, Pakistan; ^2^Liaquat University of Medical and Health Sciences, Jamshoro, Pakistan; ^3^Liaquat College of Medicine and Dentistry, Karachi, Pakistan; ^4^Atish Dipankar University of Science and Technology, Dhaka, Bangladesh; ^5^Voice of Doctors Research School, Dhaka, Bangladesh

**Keywords:** measles, measles virus, MEV, measles outbreak control, USA

## Abstract

Measles, a highly contagious respiratory illness caused by the measles virus (MeV), poses significant global and national public health challenges despite advancements in vaccination efforts. Though measles was declared eliminated in the United States in 2000, recent years have seen a resurgence of cases, particularly in under-vaccinated communities. This resurgence is compounded by factors such as vaccine hesitancy, the impact of the COVID-19 pandemic on immunization rates, and international travel introducing new cases from endemic regions. This paper examines the epidemiology and recent measles outbreaks in the United States, focusing on the significant rise in cases from 2020 to 2024. The analysis highlights the importance of maintaining high vaccination coverage, particularly in vulnerable populations, and explores the challenges of managing outbreaks. The study also reviews the pathophysiology, clinical presentation, diagnosis, and treatment of measles, emphasizing the role of prevention and control measures, including the MMR vaccine, public health interventions, and international cooperation in addressing this persistent threat.

## Introduction

Measles, a highly contagious respiratory illness, spreads primarily through airborne droplets released when an infected person coughs, sneezes, or through contact with contaminated surfaces. After exposure, symptoms usually develop within 10 to 14 days (1). Although measles was declared eliminated in the United States in 2000, the country continues to face challenges due to cases imported from other regions. This issue came to the forefront in 2019 when two significant outbreaks occurred in under-vaccinated communities in New York City, threatening the nation’s measles elimination status (2). Despite the efforts to maintain high vaccination rates, these outbreaks highlighted the ongoing risk posed by gaps in vaccine coverage.

### Etiology

Measles is caused by the measles virus (MeV), which belongs to the family Paramyxoviridae. MeV is a single-stranded RNA virus with a spherical shape coated with an envelope composed of structural proteins. It contains eight proteins, six structural, including hemagglutinin, fusion, nucleocapsid, matrix, phosphoprotein, and large protein. The hemagglutinin protein binds the virus to the host cell receptor (CD46). The fusion protein facilitates the viral envelope’s attachment to the host cell membrane and their neighboring cell, forming a multinucleated cell (3). Initially, the virus inhaled from airborne droplets or fine aerosols first targets dendritic cells, lymphocytes, and alveolar macrophages in the respiratory tract of a susceptible host. The virus initially reproduces in the epithelial cells of the respiratory tract, followed by dissemination to the local and other lymphatic sites through the bloodstream (4).

### Signs and symptoms

The incubation period for measles ranges from 7 to 21 days, with an average of 13 days (5). Measles infection occurs in two phases: prodromal and exanthematous. The prodromal phase begins 10 to 12 days after exposure, presenting with fever above 101°F, malaise, anorexia, coryza, conjunctivitis, cough, and Koplik’s spots (small white lesions on the buccal mucosa). Koplik’s spots appear in 60–70% of patients and typically last 12–72 h. The exanthematous phase follows, characterized by a red, bumpy rash that gradually coalesces and then turns coppery brown before scaling. The rash appears in a craniocaudal pattern, progressing and disappearing similarly (4, 6).

### Diagnosis

Differentiating measles from other conditions like rubella, roseola, chickenpox, erythema infectiosum (parvovirus B19) and scarlet fever is crucial. Diagnosis is confirmed by detecting IgM antibodies in serum or plasma specific to the measles virus. These antibodies are a susceptible and specific test for detecting measles and typically peak four days after the onset of the rash (7). Antigen-specific IgG antibody synthesis occurs swiftly after IgM production and typically lasts for an extended time, often throughout the individual’s life. As a result, serological detection of IgM or IgM plus IgG suggests current contact with the pathogen. Still, just IgG indicates infection at an uncertain time in the past. Because most persons in the United States have been immunized and are immune to measles, IgM is typically found in isolated cases. Without contact with a known case, detecting IgM alone is likely to be false positive reactivity rather than actual measles infection (8). RT-PCR (reverse transcriptase polymerase chain reaction) is also helpful for detecting the virus in blood, urine, nasopharyngeal, throat, or nasal samples before the appearance of IgM antibodies in serum. Moreover, it enables the genotyping of the measles virus, which is valuable for monitoring the importation and transmission of the virus (4, 7).

### Treatment

There is no specific antiviral treatment for measles; however, the MMR (measles, mumps, and rubella) vaccine remains the most effective preventive measure. The recommended age for children for vaccination is 12 months to 12 years. Post-exposure prophylaxis (PEP) with the MMR vaccine is strongly recommended for individuals with unknown vaccination status or those known to be unvaccinated. The window period for PEP is 72 h (9).

### Prevention and control

Inadequate vaccination is a primary cause of measles outbreaks in the United States. To combat this, it is crucial to ensure over 95% vaccination coverage with both doses of the measles vaccine, including catch-up vaccinations for those who missed earlier doses. Travelers entering and leaving the U.S. should be up-to-date with MMR vaccinations. Not vaccinated travelers should reconsider travel to regions with endemic measles or implement extra preventive measures when undertaking such travel. High-quality surveillance is necessary to quickly identify, report, and isolate potential cases in environmentally isolated rooms until the infectious period ends. Healthcare workers treating suspected measles cases are at an increased risk of infection. Therefore, using N-95 respirators, gloves, and pre-exposure prophylaxis is recommended.

The following recommendations are advised for patients exposed to measles without immunity based on clinical circumstances: In pediatric patients, immune globulin should be administered to infants aged 0–5 months within six days of exposure to measles. For infants aged 6–11 months, either immune globulin within six days or the measles vaccine within 72 h is recommended. Unvaccinated children over 12 months should receive the measles vaccine within 72 h of exposure, with the vaccine being preferred over immune globulin (10).

Pregnant women without immunity should receive immune globulin since the MMR vaccine is contraindicated during pregnancy. Immunocompromised patients should be administered immune globulin regardless of their immune or vaccination status. Healthcare workers who are unvaccinated and without laboratory evidence of immunity or confirmed disease should receive two doses of the measles vaccine. In cases of exposure, healthcare workers without immunity should be isolated from the workplace from day 5 to day 21 post-exposure to prevent the spread of the disease (11).

Public awareness should be raised about the potential complications of measles and the benefits of vaccination, particularly to counteract misinformation that incorrectly links the MMR vaccine to autism (12).

### Epidemiology and outbreak of measles in the USA

Measles cases have dramatically increased recently, with over 500,000 cases reported to the World Health Organization (WHO) in 2019 (13). Beginning in late 2018, two closely related outbreaks were reported within Orthodox Jewish communities in New York City (14). These outbreaks highlighted the ongoing risk posed by under-vaccinated communities and raised concerns about the United States’ measles elimination status.

From 2021 to 2022, 170 measles cases were reported, with 133 cases (78%) linked to distinct outbreaks. Of these, 47 cases (96%) in 2021 occurred among Afghan evacuees temporarily housed at U.S. military bases during Operation Allies Welcome. In 2022, 86 cases (71%) were associated with an outbreak in central Ohio (2). However, only a single outbreak, reported in Chicago in 2024, has been directly linked to a migrant facility at the U.S. southern border (15).

These events emphasize the potential for isolated importations contributing to localized outbreaks in vulnerable settings.

**Figure 1:**
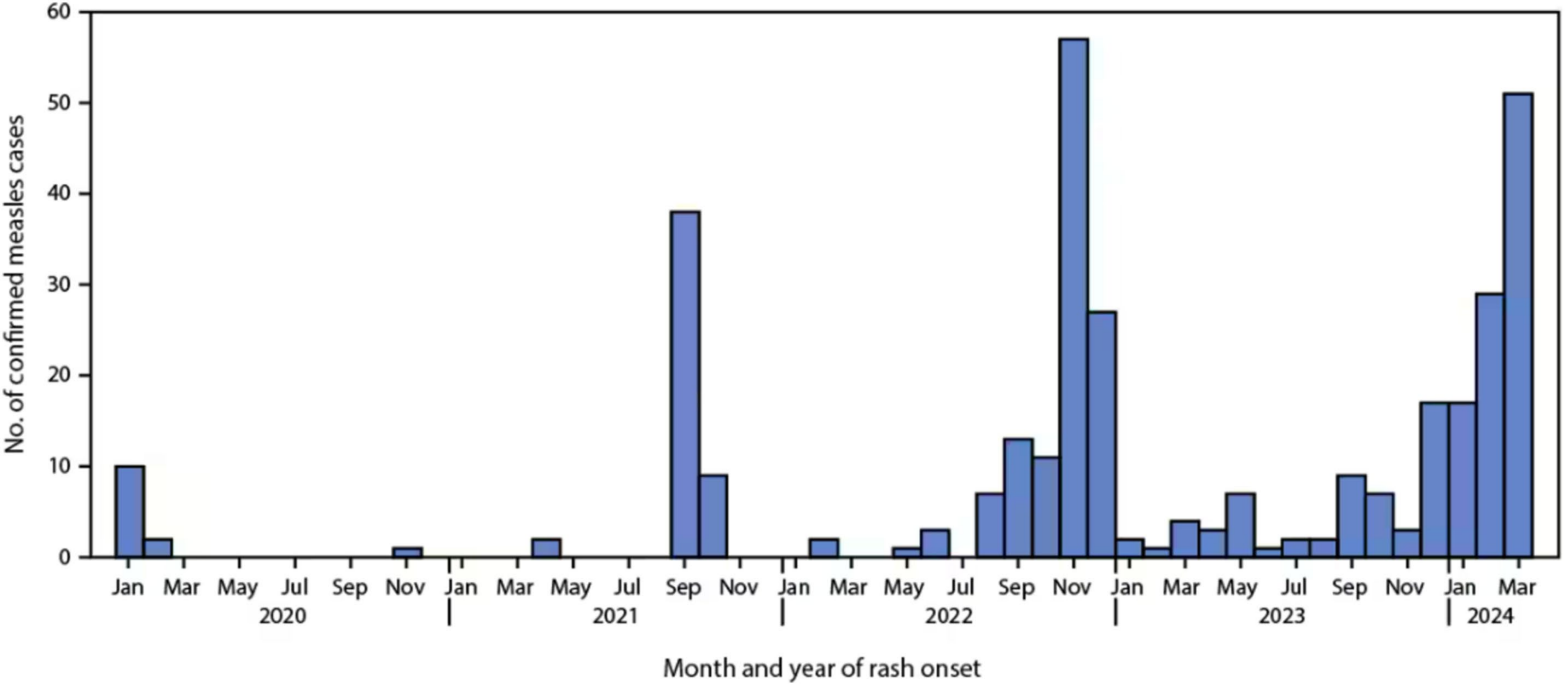
Confirmed measles cases by month of rash onset (*N* = 338) — United States, January 1, 2020–March 28, 2024. Data provided by the Center for Disease Control and Prevention (CDC) (2).

From January 1, 2020, to March 28, 2024, 338 measles cases were reported in the U.S., with 97 cases during the most recent outbreak in the first quarter of 2024 (Figure 1). Nearly all these cases involved individuals who were either unvaccinated or had unknown vaccination statuses. Despite these outbreaks, as of the end of 2023, the U.S. maintained its measles elimination status. The global rise in measles incidence and decreasing vaccination coverage have heightened the risk of importation into U.S. communities, as observed in the first quarter of 2024 (16).

As of November 21, 2024, a total of 280 measles cases had been reported in 32 states: Arizona, California, District of Columbia, Florida, Georgia, Idaho, Illinois, Indiana, Louisiana, Maryland, Massachusetts, Michigan, Minnesota, Missouri, New Hampshire, New Jersey, New Mexico, New York City, New York State, North Carolina, Ohio, Oklahoma, Oregon, Pennsylvania, South Carolina, South Dakota (17).

**Figure 2:**
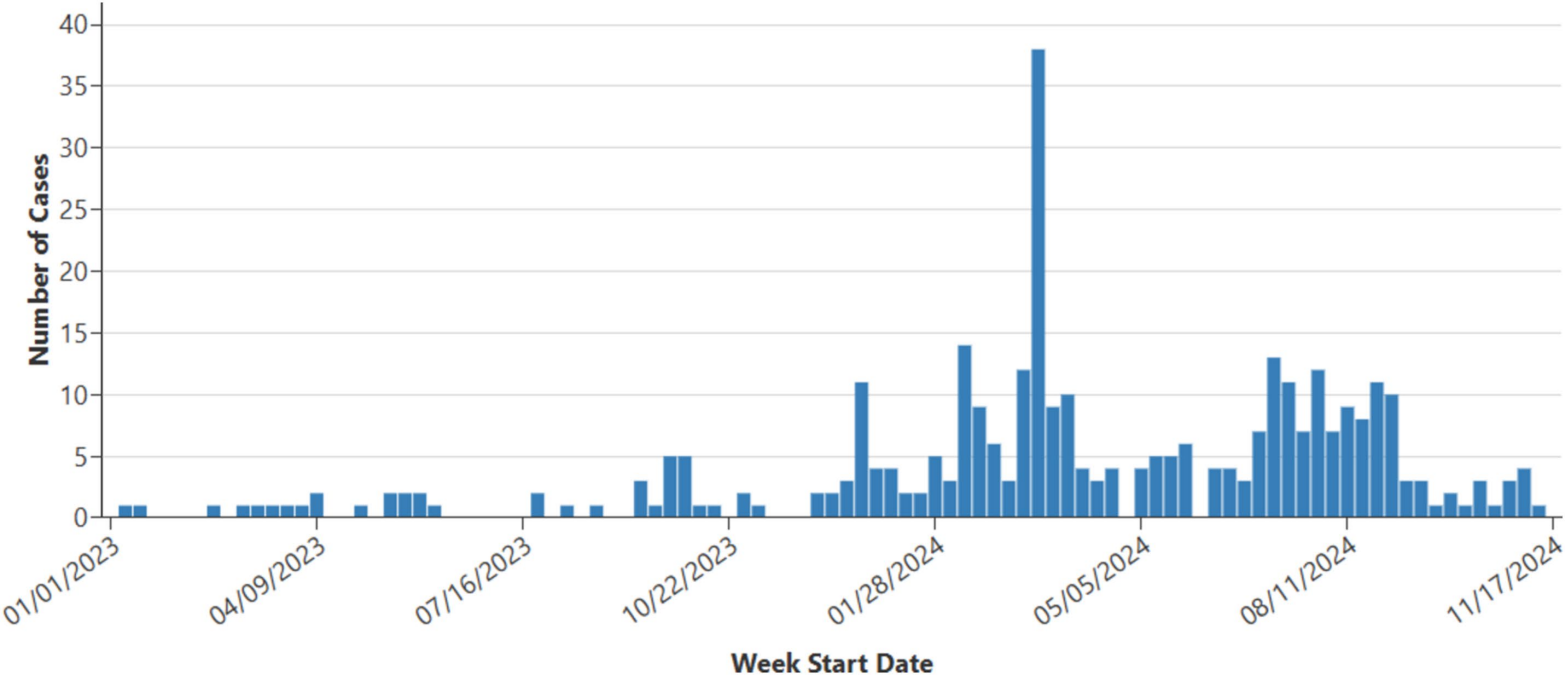
Weekly measles cases 2023–2024, data provided by Center for Disease Control and Prevention (CDC) (17).

In 2024, 16 measles outbreaks are reported, accounting for 70% (197 of 280) of all cases this year. This represents a huge increase from 2023, when just 4 outbreaks were reported, with 49% (29 out of 59) of outbreaks-related cases (Figure 2). Similar to previous years, most cases in 2024 occurred among unvaccinated individuals under 20 years old, shelter residents, or those linked to importations (2).

### Challenges encountered

Managing measles outbreaks in the U.S. faces several key challenges. Vaccine hesitancy, often fueled by misinformation about vaccine safety, has made specific communities particularly vulnerable to outbreaks. The COVID-19 pandemic caused significant disruptions in global immunization and disease surveillance efforts. With immunization services being suspended and vaccination rates dropping worldwide, millions of children were left vulnerable to preventable diseases, such as measles. These setbacks have increased the risk of outbreaks and undermined progress in controlling vaccine-preventable illnesses. Additionally, international travel continues introducing measles cases into the U.S., particularly from regions where the disease remains endemic. This global resurgence and the strain on public health resources needed to contain outbreaks complicate efforts to manage the disease. Legal and policy barriers, such as non-medical exemptions for vaccinations in some states, hinder efforts to maintain high vaccination rates (18).

In addressing these challenges, it is essential to prioritize sensitive and robust disease surveillance systems capable of detecting and responding to cases promptly. Effective communication with public health authorities is critical to facilitate timely case investigations, identify potential transmission chains, and implement appropriate interventions. Additionally, it requires a comprehensive approach, including public education, stricter vaccination policies, and improved healthcare access to prevent and quickly contain outbreaks (19).

### Global outbreak

Measles remains a global health problem, especially in Europe and developing countries. Nearly 95,000 measles cases were reported worldwide in 2024, with projections indicating the total number could match or exceed the previous year’s figures. On January 19, 2024, the UK Health and Security Agency declared a national incident in response to the escalating measles cases nationwide, cautioning that the disease could further spread. Despite the UK achieving measles elimination status in 2021, since October 2023, there have been 216 confirmed cases and 103 probable cases, primarily affecting children under 10 in the West Midlands. Between January and October 2023, over 30,000 cases were reported in the WHO European region. Outbreaks are also ongoing in Yemen (25,216 cases), India (14,927 cases), Kazakhstan (12,985 cases), and Ethiopia (11,227 cases) with provisional data from early December 2023 indicating thousands of cases in each of these countries (20, 21).

## Conclusion

Measles continues to be a significant global public health concern in the United States despite previous progress toward its elimination. The recent increase in measles cases, particularly among under-vaccinated populations, underscores the need for sustained efforts to maintain high vaccination coverage and address the challenges posed by vaccine hesitancy and global health disruptions. Effective management of measles outbreaks requires a multifaceted approach, including robust vaccination programs, public education, improved surveillance, and international collaboration. The resurgence of measles serves as a reminder of the importance of vigilance in public health practices and the ongoing need to protect vulnerable populations from vaccine-preventable diseases.

## Data Availability

The original contributions presented in the study are included in the article/supplementary material, further inquiries can be directed to the corresponding author.
